# High burden of kidney involvement in neuronal intranuclear inclusion disease

**DOI:** 10.3389/fneur.2026.1905689

**Published:** 2026-08-11

**Authors:** Zhenxian Hu, Hongfei Tai, An Wang, Yi Zhou, Hua Pan, Zaiqiang Zhang

**Affiliations:** 1 Department of Neurology, Beijing Tiantan Hospital, Capital Medical University, Beijing, China; 2 China National Clinical Research Center for Neurological Diseases, Beijing, China

**Keywords:** Intranuclear inclusions, kidney involvement, neuronal intranuclear inclusion disease, *NOTCH2NLC* GGC repeat, urinary renal function parameters

## Abstract

**Background:**

Neuronal intranuclear inclusion disease (NIID) is a rare neurodegenerative disorder caused by *NOTCH2NLC* GGC repeat expansions. Renal involvement in NIID is not well understood.

**Methods:**

We conducted a cross-sectional study of 96 genetically confirmed NIID patients. Renal function was comprehensively assessed using the estimated glomerular filtration rate (eGFR) derived from the 2021 Chronic Kidney Disease Epidemiology Collaboration creatinine equation (CKD-EPI 2021; hereafter EPI-eGFR) and a panel of five urinary biomarkers (microalbumin, transferrin, immunoglobulin G, α1-microglobulin, and α2-macroglobulin). Clinical features, GGC repeat number, and skin intranuclear inclusion count were analyzed for their association with renal function. Renal biopsies from two patients with pre-neurological proteinuria were examined for intranuclear inclusions.

**Results:**

Reduced EPI-eGFR (<90 mL/min/1.73 m^2^) was present in 20.8% (20/96) of patients. Among 44 patients with normal eGFR who underwent urinary testing, 63.6% (28/44) had abnormal urinary biomarkers. Urinary microalbumin, transferrin, immunoglobulin G and α1-microglobulin were significantly elevated in the early renal dysfunction group when eGFR remained normal (all *p* < 0.001). Among individual biomarkers, urinary microalbumin showed the best discrimination for early renal involvement (AUC 0.905). Hypertension was independently associated with reduced eGFR (OR 3.96, 95% CI: 1.16–13.51, *p* = 0.028). GGC repeat number showed no overall correlation with eGFR, nor did skin inclusion burden (all *p* > 0.05). However, in patients with normal renal function, higher GGC repeat number correlated with lower eGFR (*ρ* = −0.528, *p* = 0.035). Renal biopsies from two patients with pre-neurological proteinuria revealed characteristic p62-positive intranuclear inclusions in tubular and glomerular cells.

**Conclusion:**

Kidney involvement is common in this hospital-based NIID cohort, with a substantial proportion showing subclinical renal impairment. Sensitive urinary biomarkers may aid early detection before eGFR declines, with microalbumin showing the highest discriminative ability. Hypertension is a potentially modifiable risk factor for reduced eGFR. Genetic burden and skin inclusion burden do not predict renal outcomes, suggesting that secondary mechanisms drive progression. For patients who present with unexplained renal impairment, pathological examination of renal biopsy specimens for intranuclear inclusions may provide critical diagnostic clues.

## Introduction

Neuronal intranuclear inclusion disease (NIID) is a rare and progressive neurodegenerative disorder. Its main neuropathological feature is the widespread presence of eosinophilic hyaline intranuclear inclusions in the nervous system (1). The discovery that expanded GGC repeats in the *NOTCH2NLC* gene cause NIID has greatly improved molecular diagnosis and opened new directions for research. It is now clear that NIID belongs to a growing spectrum of repeat expansion disorders (2, 3).

NIID has traditionally been seen as a neurological disease. Its clinical presentation is highly variable and includes dementia, encephalopathy, autonomic dysfunction, cerebellar ataxia, and peripheral neuropathy (4). However, growing evidence suggests that systemic involvement is a core feature of the disease, not an occasional finding. Skin biopsy has become a key diagnostic tool, as inclusions in sweat glands and fibroblasts offer a less invasive way to confirm the diagnosis (1). In addition, studies have reported intranuclear inclusions in other visceral organs, supporting the view that NIID is a true systemic disorder (5, 6).

Despite these advances, renal involvement in NIID remains markedly undercharacterized. Early case reports have documented inclusions in renal tubular cells, suggesting potential renal pathology (7). Nevertheless, a systematic evaluation of renal function and the spectrum of kidney injury in a defined NIID cohort is lacking. Previous cohort studies (8) did not investigate urinary markers of early kidney injury and risk factors for renal dysfunction in NIID patients. The causal link between intranuclear inclusion burden and renal dysfunction also remains largely unexplored.

In this study, we conducted a systematic cross-sectional analysis of 96 consecutively enrolled NIID patients. We aimed to fully characterize the spectrum of renal involvement in NIID, investigate how genetic burden and intranuclear inclusion burden relate to renal function, and identify risk factors for renal dysfunction.

## Methods

### Study design and participants

We conducted a monocentric, cross-sectional study at Beijing Tiantan Hospital, Capital Medical University. Consecutive patients diagnosed with NIID between June 2018 and March 2026 were assessed for eligibility. The diagnosis of NIID was confirmed according to established criteria (5), integrating clinical presentation, characteristic neuroimaging features, distinctive skin biopsy findings and genetic confirmation of a pathogenic GGC repeat expansion in the 5′-UTR of the *NOTCH2NLC* gene.

Participants were included if they (1) were aged ≥14 years; (2) had a genetically confirmed diagnosis of NIID with a pathogenic *NOTCH2NLC* GGC repeat expansion; and (3) had complete renal function data available. Patients were excluded for any of the following: (1) concurrent renal disease of a known alternative etiology; (2) acute kidney injury within the preceding 3 months; (3) pregnancy or lactation; or (4) incomplete clinical or laboratory data precluding accurate classification of renal function status. This study was approved by the Institutional Review Board of Beijing Tiantan Hospital, Capital Medical University. All participants or their legal guardians provided written informed consent prior to their inclusion in the study.

The enrolled patients underwent a comprehensive renal function evaluation. The estimated glomerular filtration (eGFR) rate was calculated using the 2021 Chronic Kidney Disease Epidemiology Collaboration creatinine-based equation (CKD-EPI 2021 creatinine equation; hereafter EPI-eGFR) (9). Urinary renal function parameters, including microalbumin, transferrin, immunoglobulin G, α1-microglobulin, and α2-macroglobulin, were also measured.

A predefined set of demographic, clinical, pathological and genetic variables was systematically collected for comparative analysis. Demographic and clinical data, including age at admission, sex, age at onset, disease duration, age at diagnosis, onset-to-diagnosis interval, body mass index (BMI), detailed clinical phenotyping (10), history of hypertension, history of diabetes, and family history, were collected primarily from structured medical records. Patients were assigned to one of five predominant clinical phenotypes, cognitive impairment, episodic neurogenic events, movement disorders, autonomic dysfunction, and neuromuscular disease (10). We acknowledge that this scheme is a deliberate clinical simplification and does not fully capture the substantial symptomatic overlap observed in many patients. Poorly controlled hypertension was defined as in-hospital blood pressure ≥ 140/90 mmHg or requirement for ≥ 2 antihypertensive agents. Additional data on healthcare monitoring and antihypertensive treatment, such as outpatient visits before admission, renal function tests before admission, the use of angiotensin receptor blockers (ARB) or angiotensin-converting enzyme inhibitors (ACEI), and regular antihypertensive treatment, were also retrieved from the same source. All biochemical parameters, including glycated hemoglobin (HbA1c), serum creatinine, blood urea nitrogen (BUN), blood albumin, qualitative urinary protein, and renal function parameters were measured by the central laboratory of a tertiary academic hospital using analytically validated methods and automated platforms. Internal and external quality control procedures were strictly implemented to ensure the accuracy and reliability of the results.

### Burden of intranuclear inclusions

The burden of intranuclear inclusions is reflected by the intranuclear inclusion count. P62-stained skin biopsy sections were examined at 200 × magnification, and five fields per sample were selected to maximize visualization of intranuclear inclusions. The inclusion count per field was calculated as the number of inclusions divided by the number of cells. The mean inclusion count across these fields was calculated for each patient. Intranuclear inclusion count was performed in 56 patients, and correlations with renal function were subsequently analysed.

### Renal histological analysis

Renal biopsy specimens were obtained from two enrolled patients who had developed proteinuria before exhibiting neurological symptoms related to NIID. These specimens, originally acquired from external nephrology departments, underwent thorough histological analysis and reexamination to detect the presence of intranuclear inclusions.

### Genetic data

To ensure accurate variant interpretation, all genetic results were independently assessed by two neurologists with subspecialty expertise in inherited neurological diseases. Pathogenic validation was confirmed through this multidisciplinary review. For all the enrolled patients, GGC repeat expansions in the 5′-untranslated region of the *NOTCH2NLC* gene were analysed using repeat-primed polymerase chain reaction (PCR). When expansions exceeded the detectable range of conventional fragment analysis, Cas9-enhanced nanopore sequencing was applied for comprehensive characterization (2).

### Data analysis

Continuous variables were tested for normality using the Shapiro–Wilk test. Normally distributed data were presented as mean (standard deviation) and compared using the independent samples t-test for two groups or one-way analysis of variance (ANOVA) for three groups. Non-normally distributed data were presented as median with interquartile range (IQR) and compared using the Mann–Whitney U test for two groups or the Kruskal–Wallis H test for three groups. Categorical data were presented as counts and percentages [N (%)], and comparisons between two groups were made using the chi-square test or Fisher’s exact test. The Bonferroni correction method was used for post-hoc pairwise comparisons among three groups. The associations of *NOTCH2NLC* GGC repeat expansion size and intranuclear inclusion count with EPI-eGFR were assessed using Pearson/Spearman correlation, simple and multiple linear regression, curve estimation (including linear, quadratic, cubic, logarithmic, power, and exponential models), and tertile-based ANOVA with linear trend tests. The variables selected were included in a binary logistic regression to identify factors associated with decreased eGFR (<90 mL/min/1.73 m^2^). A sensitivity analysis was performed to assess whether abnormal urinary biomarker frequency was affected by diabetes or hypertension. Abnormal biomarker rates between the mutually exclusive comorbidity-free and comorbidity groups were compared using Fisher’s exact test. Receiver operating characteristic (ROC) curve analysis was used to evaluate the ability of individual urinary biomarkers to discriminate patients with early renal involvement from those with normal urinary findings among patients with preserved eGFR. The area under the ROC curve (AUC) and its 95% confidence interval were estimated under the nonparametric assumption, and the optimal cutoff for each biomarker was determined as the point maximizing the Youden index (sensitivity + specificity − 1). Statistical analyses were conducted with SPSS version 26.0 (IBM Corp., Armonk, NY, USA). A two-tailed test was employed, and statistical significance was set at *p* < 0.05.

## Results

### Baseline characteristics and spectrum of renal impairment

During the study period, a total of 103 hospitalized patients clinically diagnosed with suspected NIID and meeting preliminary eligibility criteria were enrolled in our cohort. Among these, three patients were excluded due to end-stage multiple organ failure. Additionally, four patients were excluded because *NOTCH2NLC* GGC repeat expansion size could not be obtained. Consequently, a total of 96 patients were included in the final analysis, comprising 40 males (41.67%) and 56 females (58.33%). The median age of the cohort was 63.50 (IQR: 59.00–67.00) years, the median age at onset was 57.50 (IQR: 49.00–62.00) years, and the median disease duration was 5.00 (IQR: 2.00–10.00) years. Regarding clinical phenotypes, 15 patients (15.63%) presented with cognitive impairment as the predominant phenotype, 34 patients (35.42%) with episodic neurogenic events, 29 patients (30.21%) with movement disorders, 14 patients (14.58%) with autonomic dysfunction, and 4 patients (4.17%) with neuromuscular disease. Comorbidities included hypertension in 48 patients (50.00%) and diabetes mellitus in 24 patients (25.00%). A family history of NIID was present in 49 patients (51.04%).

Among the 96 enrolled patients, 20 (20.83%) had an EPI-eGFR < 90 mL/min/1.73 m^2^, while 76 (79.17%) had an EPI-eGFR ≥ 90 mL/min/1.73 m^2^. The renal impairment was predominantly mild. Among the 20 patients with EPI-eGFR < 90 mL/min/1.73 m^2^, 14 (14.58%) were classified as CKD stage (11) G2 (EPI-eGFR 60–89 mL/min/1.73 m^2^), 4 (4.17%) as stage G3a–b (EPI-eGFR 30–59 mL/min/1.73 m^2^), and only 2 (2.08%) as stage G4–5 (EPI-eGFR < 30 mL/min/1.73 m^2^). Among the 76 patients with normal EPI-eGFR (≥90 mL/min/1.73 m^2^), 44 underwent further urinary renal function testing for early renal impairment. Of these, 28 (63.64%) showed abnormalities in urinary renal function parameters. When combining the five urinary renal function parameters, the proportion of renal impairment further increased to approximately 75% (48/64).

### Comparison of clinical characteristics across different renal function strata

Patients were stratified according to EPI-eGFR into the renal dysfunction group (*n* = 20, EPI-eGFR < 90 mL/min/1.73 m^2^) and normal eGFR group (*n* = 76, EPI-eGFR ≥ 90 mL/min/1.73 m^2^). No statistically significant differences were observed between the two groups in terms of age at admission, sex, BMI, age at onset, disease duration, clinical phenotype, prevalence of diabetes mellitus, HbA1c, family history of NIID, GGC repeat number, intranuclear inclusion count, or serum albumin (all *p* > 0.05) (Table 1). However, the prevalence of hypertension was significantly higher in the renal dysfunction group compared with the normal eGFR group (75.00% vs. 43.42%, χ^2^ = 6.316, *p* = 0.022) (Table 1). As expected, serum creatinine levels were significantly elevated in the renal dysfunction group (median 92.55 μmol/L, IQR: 71.70–109.85) compared with the normal eGFR group (median 55.25 μmol/L, IQR: 48.05–62.60) (*p* < 0.001). Similarly, BUN levels were also significantly higher in the renal dysfunction group (median 6.25 mmol/L, IQR: 4.98–7.83) than in the normal eGFR group (median 5.05 mmol/L, IQR: 4.28–5.83) (*p* = 0.004) (Table 1).

**Table 1:** Comparison of clinical characteristics between normal eGFR group and renal dysfunction group.

Item	Normal eGFR group (*n* = 76)	Renal dysfunction group (*n* = 20)	Z/χ^2^/t	*p*
Age at admission, years, M (Q1, Q3)	63.00 (59.00, 66.00)	65.00 (60.75, 69.00)	Z = −1.520	0.129
Male, *n* (%)	31 (40.79%)	9 (45.00%)	χ^2^ = 0.115	0.734
BMI, kg/m^2^, mean (SD)	22.68 (3.42)	23.80 (2.90)	t = −1.306	0.195
Age of onset, years, M (Q1, Q3)	58.00 (49.00, 62.00)	56.50 (49.75, 60.75)	Z = 0.045	0.968
Disease duration, years, M (Q_1_, Q_3_)	5.00 (2.00, 10.00)	6.50 (4.00, 10.00)	Z = −0.952	0.342
Cognitive impairment, *n* (%)	11 (14.47%)	4 (20.00%)	χ^2^ = 0.367	0.508
Episodic neurogenic event, *n* (%)	28 (36.84%)	6 (30.00%)	χ^2^ = 0.324	0.569
Movement disorder, *n* (%)	24 (31.58%)	5 (25.00%)	χ^2^ = 0.325	0.569
Autonomic dysfunction, *n* (%)	10 (13.16%)	4 (20.00%)	χ^2^ = 0.595	0.481
Neuromuscular disease, *n* (%)	3 (3.95%)	1 (5.00%)	χ^2^ = 0.044	> 0.999
Hypertension, *n* (%)	33(43.42%)	15 (75.00%)	χ^2^ = 6.316	**0.022**
Diabetes, *n* (%)	17 (22.37%)	7(35.00%)	χ^2^ = 1.347	0.246
HbA1c,%, M (Q_1_, Q_3_)	5.90 (5.50, 6.40)	6.15 (5.60, 6.35)	Z = −0.603	0.549
Family history, *n* (%)	42 (55.26%)	7 (35.00%)	χ^2^ = 2.602	0.107
Serum creatinine, μmol/L, M (Q_1_, Q_3_)	55.25 (48.05, 62.60)	92.55 (71.70, 109.85)	Z = −6.090	**<0.001**
BUN, mmol/L, M (Q_1_, Q_3_)	5.05 (4.28, 5.83)	6.25 (4.97, 7.83)	Z = −2.851	**0.004**
Serum albumin,g/L	38.39 ± 3.44	36.88 ± 5.28	t = 1.543	0.126
GGC repeat number, M (Q_1_, Q_3_)	115.00 (102.00, 124.25)	113.00 (101.25, 125.25)	Z = 0.397	0.695
	*n* = 46	*n* = 10		
Intranuclear inclusion count, mean (SD)	0.04 ± 0.01	0.06 ± 0.03	t = −1.861	0.068

M (Q1, Q3), median (interquartile range); BMI, body mass index; SD, standard deviation; HbA1c, glycated hemoglobin; GGC repeat number, nubmber of *NOTCH2NLC* GGC repeat expansion; BUN, Blood urea nitrogen. Bold values indicate statistical significance (*p* < 0.05).

Among the 76 patients with normal EPI-eGFR (≥90 mL/min/1.73 m^2^), 44 underwent urinary renal function testing and were further stratified into the normal renal function group (*n* = 16) and the early renal dysfunction group (*n* = 28) based on the presence of urinary abnormalities. These two groups were then compared alongside the previously defined renal dysfunction group (*n* = 20), resulting in three groups for analysis: normal renal function (*n* = 16), early renal dysfunction (*n* = 28), and renal dysfunction (*n* = 20). No statistically significant differences were observed among the three groups in terms of age at admission, sex, BMI, age at onset, disease duration, clinical phenotype, prevalence of diabetes mellitus, HbA1c, GGC repeat number, intranuclear inclusion count, or serum albumin (all *p* > 0.05). Regarding clinical phenotype, the autonomic dysfunction-predominant subtype was observed in 20% (4/20) of patients in the renal dysfunction group, 17.86% (5/28) in the early renal dysfunction group, and 6.25% (1/16) in the normal renal function group (*p* = 0.710). However, significant differences were found in the prevalence of hypertension (*p* = 0.049) and family history positivity rate (*p* = 0.040) among the three groups (Table 2). Post-hoc pairwise comparisons revealed that the prevalence of hypertension was significantly higher in the renal dysfunction group compared with the early renal dysfunction group (*p* < 0.05), while no significant difference was observed between the normal renal function group and the early renal dysfunction group. Regarding family history, the positivity rate was significantly higher in the early renal dysfunction group compared with the renal dysfunction group (p < 0.05), whereas no significant difference was found between the normal renal function group and the renal dysfunction group (Table 2).

**Table 2:** Comparison of clinical and laboratory parameters across different stages of renal dysfunction.

Item	Normal renal function group (*n* = 16)	Early renal dysfunction group (*n* = 28)	Renal dysfunction group (*n* = 20)	H/χ^2^/F	*p*
Demographics					
Age at admission, years, M (Q_1_, Q_3_)	65.00 (61.75, 66.25)	62.50 (59.00, 67.00)	65.00 (60.75, 69.00)	H = 1.434	0.448
Male, *n* (%)	5 (31.25%)	12 (42.86%)	9 (45.00%)	χ^2^ = 0.800	0.670
BMI, kg/m^2^, mean (SD)	22.84 (3.96)	22.77 (3.54)	23.80 (2.90)	*F* = 0.557	0.576
Age of onset, years, M (Q_1_, Q_3_)	56.00 (48.75, 60.25)	59.50 (48.75, 62.00)	56.50 (49.75, 60.75)	H = 0.723	0.697
Disease duration, years, M (Q_1_, Q_3_)	5.00 (3.00, 16.25)	5.50 (2.75, 10.00)	6.50 (4.00, 10.00)	H = 0.251	0.882
Comorbidity					
Hypertension, *n* (%)	9 (56.25%)	11 (39.29%)	15 (75.00%)^†^	χ^2^ = 6.026	**0.049**
Diabetes, *n* (%)	5 (31.25%)	6 (21.43%)	7 (35.00%)	χ^2^ = 1.166	0.558
HbA1c,%, M (Q_1_, Q_3_)	6.20 (5.55, 6.65)	5.60 (5.50, 6.10)	6.15 (5.60, 6.35)	H = 2.753	0.252
Kidney function parameters					
Serum creatinine, μmol/L, M (Q_1_, Q_3_)	51.10 (46.85, 62.60)	54.15 (48.03, 61.75)	92.55 (71.70, 109.85) ^†‡^	H = 32.375	**<0.001**
eGFR, mL/min/1.73m^2^, M (Q_1_, Q_3_)	102.17 (97.59, 105.99)	102.49 (100.21, 104.19)	75.22 (56.41, 82.73) ^†‡^	H = 40.618	**<0.001**
Positive for urine protein (qualitative), n (%)	0 (0.00%) ^†^	11 (39.29%)	14 (70.00%) ^‡^	χ^2^ = 18.299	**<0.001**
Urinary biomarkers			*n* = 11		
Urinary Microalbumin, mg/L, M (Q_1_, Q_3_)	7.00 (5.00, 9.00) ^†^	73.50 (22.25, 182.50)	483.44 (34.50, 599.92) ^†‡^	H = 27.282	**<0.001**
Urinary transferrin, mg/L, M (Q_1_, Q_3_)	0.00 (0.00, 0.05) ^†^	3.72 (0.26, 11.86)	51.34 (7.35, 94.04) ^†‡^	H = 24.089	**<0.001**
Urinary immunoglobulin G, mg/L, M (Q_1_, Q_3_)	0.36 (0.00, 1.66) ^†^	14.40 (2.98, 59.55)	86.86 (21.28, 215.25) ^†‡^	H = 24.190	**<0.001**
Urinary α1-microglobulin, mg/L, M (Q_1_, Q_3_)	1.26 (0.00, 3.26) ^†^	10.50 (7.30, 16.73)	15.07 (5.12, 37.32) ^†‡^	H = 18.479	**<0.001**
Urinary α2-macroglobulin, mg/L, M (Q_1_, Q_3_)	0.00 (0.00, 0.71) ^†^	1.55 (0.63, 3.22)	0.00 (0.00, 1.14)	H = 9.611	**0.008**
Others					
Family history, *n* (%)	8 (50.00%)	20 (71.43%)	7 (35.00%) ^†^	χ^2^ = 6.437	**0.040**
GGC repeat number, M (Q_1_, Q_3_)	114.50 (105.00, 128.75)	110.50 (101.75, 122.25)	113.00 (101.25, 125.25)	H = 0.860	0.650
	*n* = 11	*n* = 12	*n* = 10		
Intranuclear inclusion count, M (Q_1_, Q_3_)	0.04 (0.04, 0.05)	0.04 (0.03, 0.05)	0.05 (0.04, 0.08)	H = 1.489	0.475

M (Q1, Q3): median (interquartile range); BMI, body mass index; SD, standard deviation; HbA1c, glycated hemoglobin; GGC repeat number, number of *NOTCH2NLC* GGC repeat expansion; BUN, Blood urea nitrogen.^†^ Indicates a comparison with the early renal dysfunction group, *p* < 0.05; ^‡^ Indicates a comparison with the normal renal function group, *p* < 0.05. Bold values indicate statistical significance (*p* < 0.05).

**Figure 1:**
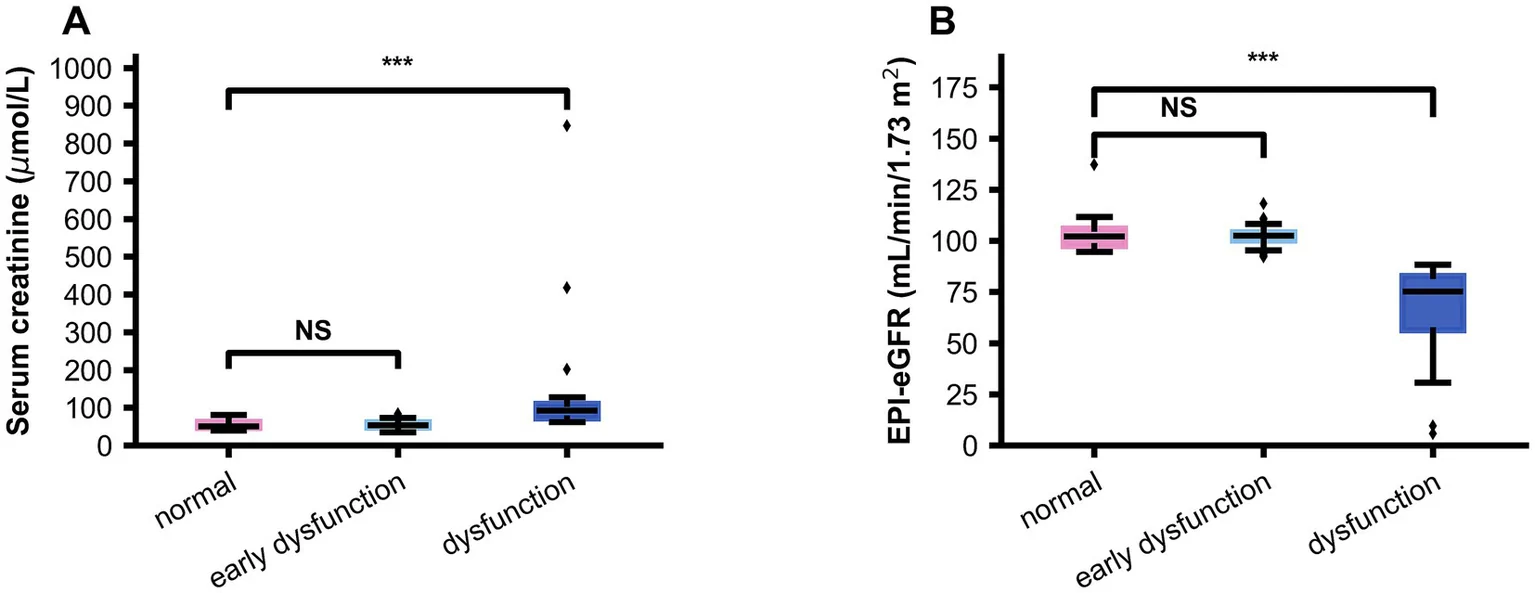
Comparison of serum creatinine **(A)** and eGFR **(B)** across renal function groups. **(A)** Serum creatinine levels showed no significant difference between the normal renal function group and the early renal dysfunction group (*p* > 0.05). **(B)** eGFR also did not differ significantly between these two groups (*p* > 0.05). In contrast, both serum creatinine and eGFR were significantly different in the renal dysfunction group compared to the other two groups. Normal: normal renal function group; early dysfunction: early renal dysfunction group; dysfunction: renal function group; NS: not significant difference; *** indicates *p* < 0.001.

Notably, no significant difference in serum creatinine levels was observed between the normal renal function group (median 51.10 μmol/L, IQR: 46.85–62.60) and the early renal dysfunction group (median 54.15 μmol/L, IQR: 48.03–61.75) (*p* > 0.05) (Figure 1A). Similarly, EPI-eGFR did not differ significantly between the normal renal function group (median 102.17 mL/min/1.73 m^2^, IQR: 97.59–105.99) and the early renal dysfunction group (median 102.49 mL/min/1.73 m^2^, IQR: 100.21–104.19) (*p* > 0.05) (Figure 1B).

### Profile of urinary biomarkers across renal function strata

**Figure 2:**
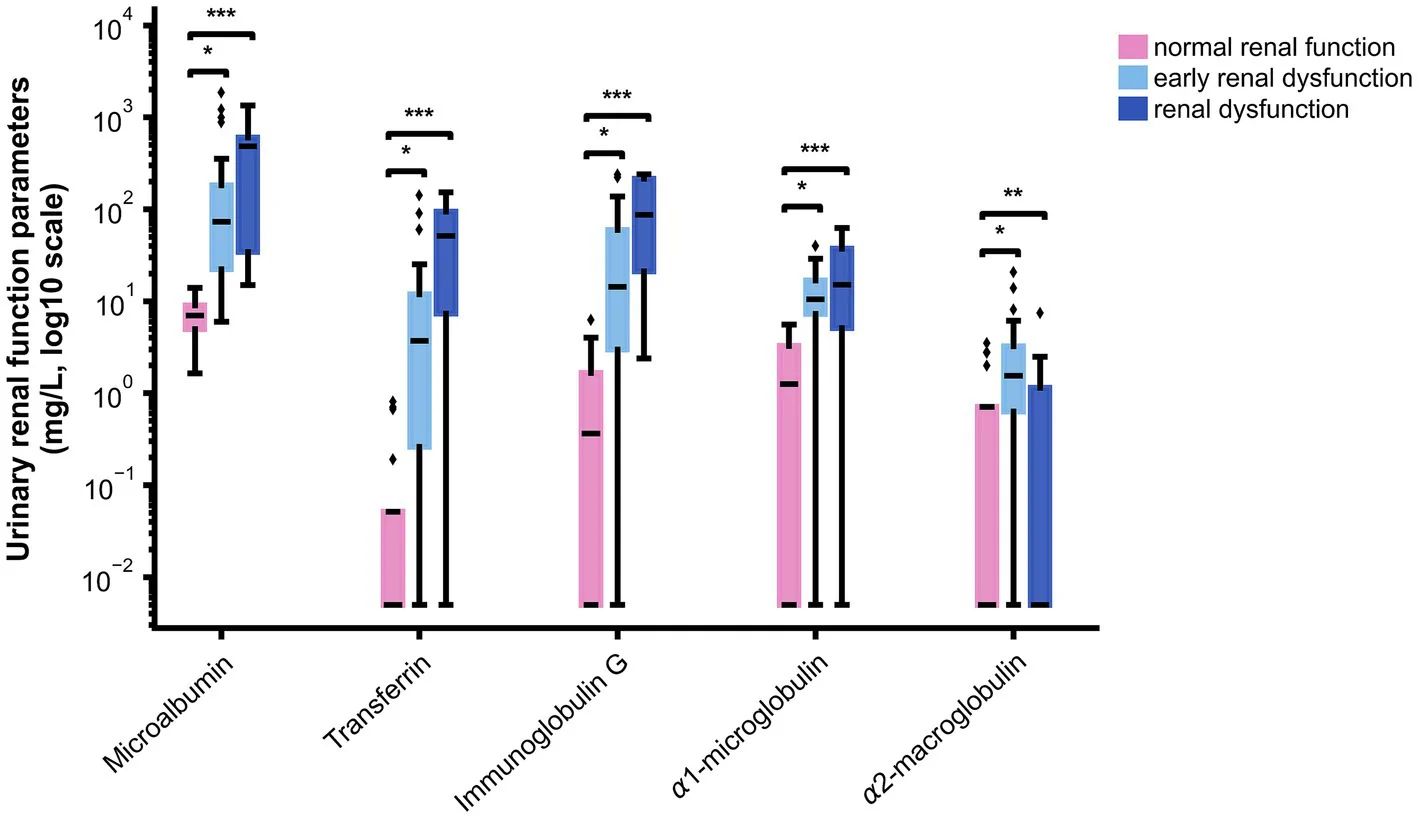
Urinary biomarker levels across normal, early dysfunction, and dysfunction groups. Levels of microalbumin, transferrin, immunoglobulin G (IgG), and α1-microglobulin increased stepwise across the three groups (all *p* < 0.001). The most pronounced median increases were observed for microalbumin, transferrin, and IgG. In contrast, although α2-macroglobulin levels differed significantly among groups (*p* = 0.008), they did not distinguish the normal renal function group from the renal dysfunction group (*p* > 0.05). *** indicates *p* < 0.001; ** indicates *p* < 0.01; * indicates *p* < 0.05.

Urinary renal function testing was performed in 44 patients from the normal eGFR group and 11 patients from the renal dysfunction group. The urinary microalbumin concentration was 7.00 (IQR: 5.00–9.00) mg/L in the normal renal function group, 73.50 (IQR: 22.25–182.50) mg/L in the early renal dysfunction group, and 483.44 (IQR: 34.50–599.92) mg/L in the renal dysfunction group. Urinary transferrin levels were 0.00 (IQR: 0.00–0.05) mg/L in the normal renal function group, 3.72 (IQR: 0.26–11.86) mg/L in the early renal dysfunction group, and 51.34 (IQR: 7.35–94.04) mg/L in the renal dysfunction group. Urinary immunoglobulin G levels were 0.36 (IQR: 0.00–1.66) mg/L, 14.40 (IQR: 2.98–59.55) mg/L, and 86.86 (IQR: 21.28–215.25) mg/L, respectively (Table 2). Significant differences among the three groups were observed for microalbumin, transferrin, immunoglobulin G, and α1-microglobulin (all *p* < 0.001). The largest median increases across the groups were seen for microalbumin, transferrin, and immunoglobulin G (Figure 2). Although urinary α2-macroglobulin levels differed significantly among the three groups (*p* = 0.008), they remained low across all groups, generally within the normal range, and failed to distinguish the normal renal function group from the renal dysfunction group (*p* > 0.05) (Table 2).

To evaluate whether the high prevalence of urinary biomarker abnormalities in patients with preserved eGFR could be largely attributed to hypertension or diabetes, we performed a sensitivity analysis restricted to the 44 patients with normal eGFR who underwent urinary testing (Table 3). Among patients without diabetes, the abnormal biomarker rate was 66.67% (22/33); among those without hypertension, the rate was 70.83% (17/24); among those without poorly controlled hypertension, the rate was 68.75% (22/32) and among patients free of both comorbidities, the rate was 76.19% (16/21). When patients with poorly controlled hypertension were excluded, the rate among the remaining comorbidity-free patients was 76.0% (19/25) (Table 3, Part A). In all subgroups, the abnormal rate remained at or above 64%. To further test this, two mutually exclusive groups were compared directly (Table 3, Part B). When any hypertension was considered, the abnormal rate was 76.19% (16/21) in the comorbidity-free group and 52.17% (12/23) in the comorbidity group (Fisher’s exact test, *p* = 0.125). When only poorly controlled hypertension was considered, the corresponding rates were 76.00% (19/25) and 47.37% (9/19) (*p* = 0.065). The two groups did not differ significantly in either comparison, and the abnormal rate was numerically higher, rather than lower, in the comorbidity-free group.

**Table 3:** Sensitivity analysis of abnormal urinary biomarker proportion among patients with preserved EPI-eGFR after excluding common comorbidities.

Part A: abnormal urinary biomarker proportion after stepwise exclusion			
Subgroup	*n*	Abnormal, *n*	Abnormal, %
All patients with preserved eGFR tested	44	28	63.64
Excluding diabetes	33	22	66.67
Excluding hypertension	24	17	70.83
Excluding poorly controlled hypertension	32	22	68.75
Excluding diabetes and hypertension	21	16	76.19
Excluding diabetes and poorly controlled hypertension	25	19	76.00

Part B: comorbidity-free vs comorbidity group				
Comparison	Comorbidity-free	Comorbidity group	OR (95% CI)	Fisher *p*
Diabetes and/or hypertension	16/21 (76.19%)	12/23 (52.17%)	2.93 (0.80–10.71)	0.125
Diabetes and/or poorly controlled hypertension	19/25 (76.00%)	9/19 (47.37%)	3.52 (0.97–12.73)	0.065

Abnormal, Abnormal urinary biomarker. Poorly controlled hypertension was defined as an in-hospital blood pressure ≥ 140/90 mmHg or the need for ≥ 2 antihypertensive agents. In Part B, the comorbidity-free and comorbidity groups are mutually exclusive and were compared using Fisher’s exact test. OR, odds ratio; CI, confidence interval.

**Figure 3:**
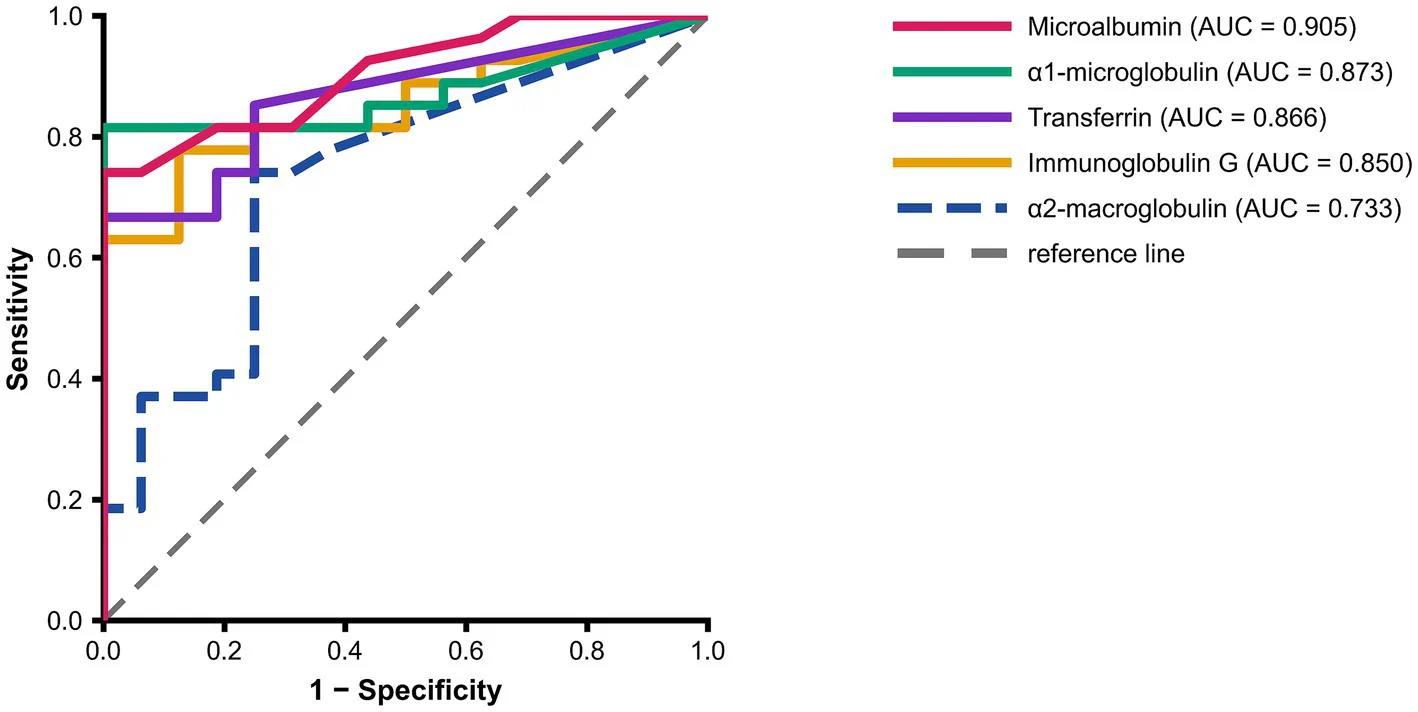
Receiver operating characteristic (ROC) curves of urinary biomarkers for detecting early renal injury in NIID patients with preserved eGFR.

To evaluate the ability of urinary biomarkers to detect early renal involvement, ROC analyses were performed for each marker among patients with preserved EPI-eGFR, comparing the early renal involvement group (*n* = 28) to the normal renal function group (*n* = 16) (Table 4, Figure 3). Among the five markers, urinary microalbumin showed the best discrimination, with an AUC of 0.905 (95% CI 0.819–0.991). The optimal cutoff was 20.0 mg/L, which gave a sensitivity of 0.741 and a specificity of 1.00. The other markers had lower AUCs: α1-microglobulin (0.873), transferrin (0.866), immunoglobulin G (0.850), and α2-macroglobulin (0.733). Individual-marker performance was reported descriptively.

**Table 4:** Discriminative performance of individual urinary biomarkers for early renal involvement among patients with preserved EPI-eGFR.

Biomarker	*n*	AUC	95% CI	Optimal cutoff	Sensitivity	Specificity
Urinary microalbumin	44	0.905	0.819–0.991	20.00 mg/L	0.741	1.000
Urinary α1-microglobulin	43	0.873	0.762–0.983	5.75 mg/L	0.815	1.000
Urinary transferrin	43	0.866	0.759–0.973	0.92 mg/L	0.667	1.000
Urinary immunoglobulin G	43	0.850	0.736–0.963	2.25 mg/L	0.778	0.875
Urinary α2-macroglobulin	43	0.733	0.576–0.889	0.40 mg/L	0.741	0.750

AUC, area under the receiver operating characteristic curve; CI, confidence interval. Because these cutoffs were derived from a single, modest-sized cohort, external validation in independent cohorts is required before clinical implementation.

### Ultrasonographic findings

Ultrasound was performed in 90 patients, including 20 in the renal dysfunction group and 70 in the normal eGFR group.

Renal ultrasound findings were unremarkable in most patients. Notable exceptions included four patients (4/20, 20.0%) in the renal dysfunction group who exhibited altered renal morphology and size on ultrasound, of whom two showed increased cortical echogenicity with loss of corticomedullary differentiation and two with CKD Stage G4–5 had renal atrophy. In the normal eGFR group, one patient with early renal dysfunction (1/70, 1.4%) had such morphology abnormalities. The difference between the two groups was statistically significant (*p* = 0.008). Hydronephrosis was observed in 5 of 70 patients (7.1%) in the normal eGFR group and in 3 of 20 patients (15.0%) in the renal dysfunction group, with no significant difference between the groups (*p* = 0.369).

Bladder ultrasound revealed a postvoid residual volume >50 mL in 21 of 70 patients (30.0%) in the normal eGFR group and in 7 of 20 patients (35.0%) in the renal dysfunction group, a difference that was not statistically significant (*p* = 0.670). Neurogenic bladder changes were observed in 21 of 70 patients (30.0%) in the normal eGFR group and in 6 of 20 patients (30.0%) in the renal dysfunction group (*p* > 0.999).

### Genetic and pathological correlations of renal function

**Figure 4:**
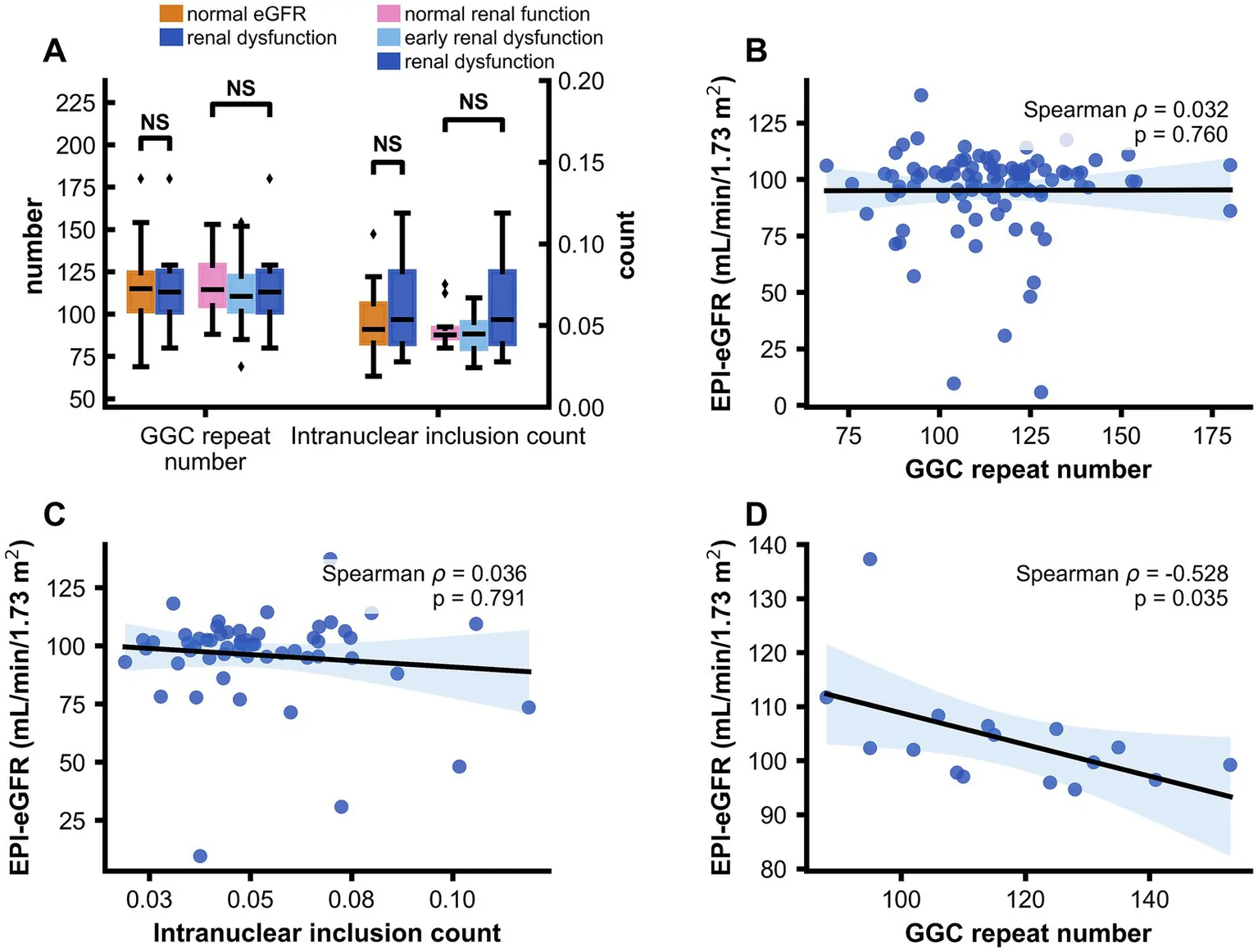
Association of *NOTCH2NLC* GGC repeat number and intranuclear inclusion burden with EPI-eGFR. **(A)** Box plots comparison of pathogenic *NOTCH2NLC* GGC repeat number revealed no significant difference across the renal function groups, either in the two-group comparison (*p* = 0.695) or in the three-group comparison (*p* = 0.650). Similarly, the intranuclear inclusion count did not differ significantly across the groups, in neither the two-group (*p* = 0.068) nor the three-group comparison (*p* = 0.475). **(B,C)** Scatter plots show the relationship between EPI-eGFR and GGC repeat number **(B)** and intranuclear inclusion count **(C)** in the overall cohort. No significant correlations were detected (Spearman correlation: GGC repeat number, *ρ* = −0.042, *p* = 0.683; inclusion count, *ρ* = 0.009, *p* = 0.947). Solid lines represent linear regression fits, with shaded areas indicating 95% confidence intervals. **(D)** Subgroup analysis in patients with normal renal function revealed a significant negative correlation between GGC repeat number and EPI-eGFR (Spearman *ρ* = −0.528, *p* = 0.035). The solid line represents the linear regression fit, with the shaded area indicating the 95% confidence interval. GGC repeat number: number of *NOTCH2NLC* GGC repeat expansion; NS: not significant difference.

Analysis of pathogenic *NOTCH2NLC* GGC repeat expansion size revealed no significant difference across the renal function groups, either in the two-group comparison (*p* = 0.695) or in the three-group comparison (*p* = 0.650) (Tables 1, 2; Figure 4A). Similarly, the intranuclear inclusion count did not differ significantly across the renal function groups, in neither the two-group (*p* = 0.068) nor the three-group comparison (*p* = 0.475) (Tables 1, 2; Figure 4A).

To further evaluate the association between EPI-eGFR and both GGC repeat number and inclusion count, Pearson correlation, Spearman rank correlation, and simple linear regression analyses were performed. All three methods yielded consistent results, showing no significant linear or monotonic association between GGC repeat number and EPI-eGFR, nor between intranuclear inclusion count and EPI-eGFR (Table 5; Figures 4B,C). Subsequent multiple linear regression, curve estimation with six functional forms (linear, quadratic, cubic, logarithmic, power, and exponential), and tertile-based ANOVA analyses revealed no statistically significant linear, nonlinear, or categorical associations between either GGC repeat number or intranuclear inclusion count and renal function as reflected by EPI-eGFR (Table 5; Supplementary Tables 1, 2).

**Table 5:** Association of GGC repeat number and intranuclear inclusion count with EPI-eGFR – multiple analytical approaches.

Item	*n*	Pearson r (*p*)	Spearman ρ (*p*)	Univariate linear regression	Multivariate linear regression*	Curve estimation^#^(best R^2^, *p*)	Tertile comparison ANOVA *p*
GGC repeat number	96	0.003 (0.976)	0.032 (0.760)	B = 0.003, *p* = 0.976	B = −0.008, *p* = 0.956	R^2^ = 0.005 (cubic), *p* = 0.924	0.870
Intranuclear inclusion count	56	−0.112 (0.412)	0.036 (0.791)	B = −106.6, *p* = 0.412	B = −130.837, *p* = 0.305	R^2^ = 0.047 (cubic), *p* = 0.470	0.654

GGC repeat number: number of *NOTCH2NLC* GGC repeat expansion.* Multivariate linear regression model included age at admission, age at onset, family history, hypertension history, GGC repeat number and intranuclear inclusion count (*n* = 56 for GGC repeat number model, *n* = 56 for intranuclear inclusion count). No significant association was found in any model. ^#^Curve estimation tested linear, quadratic, cubic, logarithmic, power, and exponential models; the best R^2^ and its *p* value are shown.

Further subgroup analysis revealed that, among patients with normal renal function, Spearman correlation analysis showed a significant negative correlation between GGC repeat number and EPI-eGFR (*ρ* = −0.528, *p* = 0.035) (Figure 4D). However, no significant correlation was observed in the early renal dysfunction group (*p* = 0.457) or in the renal dysfunction group (*p* = 0.872). In contrast, inclusion count showed no significant correlation with EPI-eGFR in any of the subgroup analyses (all *p* > 0.05).

### Factors associated with decreased eGFR

**Figure 5:**
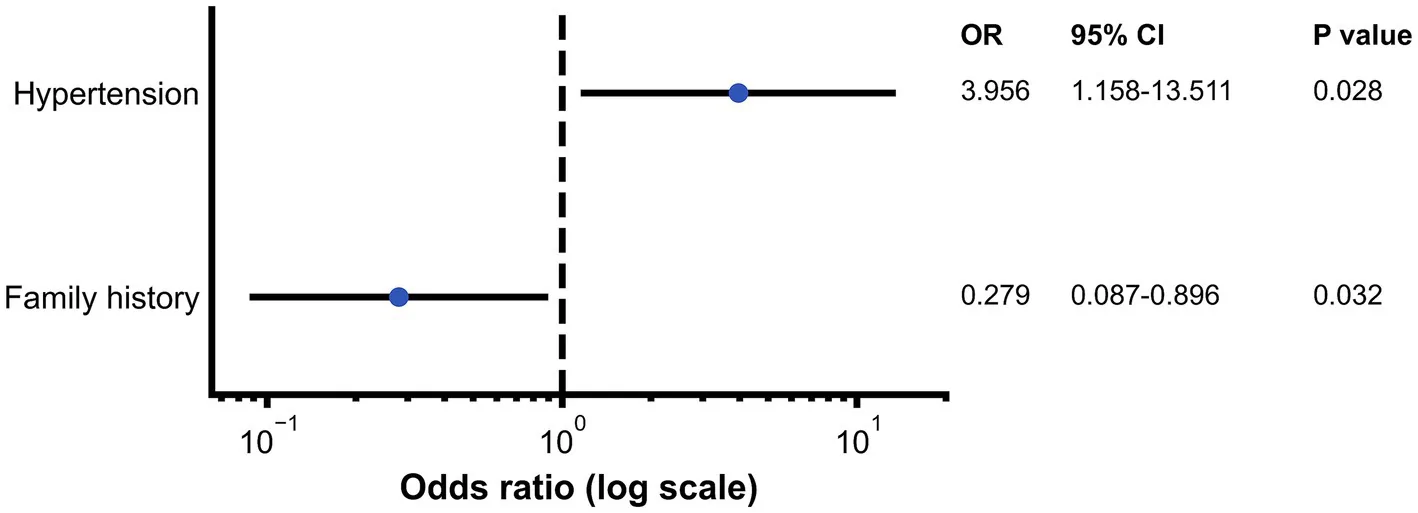
Forest plot of factors associated with decreased eGFR (<90 mL/min/1.73 m^2^). Adjusted odds ratios (*OR*s) and 95% confidence intervals (*CI*s) were displayed. Hypertension was associated with increased risk (*OR* = 3.956, 95% *CI*: 1.158–13.511, *p* = 0.028), while family history showed a protective effect (*OR* = 0.279, 95% *CI*: 0.087–0.896, *p* = 0.032). The dashed vertical line represents an *OR* of 1.

Binary logistic regression analysis was performed to identify factors associated with decreased eGFR (<90 mL/min/1.73 m^2^). The model was statistically significant (χ^2^ = 9.919, df = 2, *p* = 0.007) and demonstrated adequate goodness-of-fit (Hosmer-Lemeshow test: χ^2^ = 0.404, *p* = 0.817) (Table 6). After adjustment, hypertension was significantly associated with an increased risk of eGFR < 90 mL/min/1.73 m^2^ (OR = 3.956, 95% CI: 1.158–13.511, *p* = 0.028) (Figure 5). However, family history showed a paradoxical protective effect (OR = 0.279, 95% CI: 0.087–0.896, *p* = 0.032) (Figure 5). The overall classification accuracy was 71.9%, with a sensitivity of 45.0% and a specificity of 84.1% for predicting eGFR < 90 mL/min/1.73 m^2^.

**Table 6:** Binary logistic regression analysis for factors associated with EPI-eGFR < 90 mL/min/1.73m^2^ (*N* = 64).

Variable	β	SE	Wald	*p*	OR	95% CI
Hypertension (yes vs. no)	1.375	0.627	4.814	**0.028**	3.956	1.158–13.511
Family history (yes vs. no)	−1.275	0.595	4.600	**0.032**	0.279	0.087–0.896
Constant	−0.997	0.546	3.337	0.068	0.369	–

Hosmer-Lemeshow test *p* = 0.817; Nagelkerke R^2^ = 0.202; Overall classification accuracy = 71.9%. Bold values indicate statistical significance (*p* < 0.05).

To explore the basis of the counterintuitive protective effect of family history, we compared clinical, healthcare-monitoring, and antihypertensive-treatment indicators between family history-positive (*n* = 49) and -negative (*n* = 47) patients (Supplementary Table 3). Family history-positive patients had significantly more outpatient visits before admission (Z = −2.658, *p* = 0.008). No significant differences were found in age at onset, age at diagnosis, disease duration, onset-to-diagnosis interval, pre-admission renal function tests, or GGC repeat number (114.10 vs. 114.57, *p* = 0.907) (all *p* > 0.05). Among hypertensive patients, ARB/ACEI use (40.91% vs. 38.46%, *p* = 0.863) and regular antihypertensive treatment (63.64% vs. 65.38%, *p* = 0.900) did not differ significantly.

### Renal pathological findings in two patients with pre-neurological proteinuria

Two enrolled patients presented with unexplained substantial proteinuria (2.77 and 2.9 g/24 h, respectively) 4–5 years prior to their neurological diagnosis. Initial renal biopsies obtained during the diagnostic workup revealed membranous nephropathy and focal segmental glomerulosclerosis, respectively, with no definitive etiology identified at that time.

**Figure 6:**
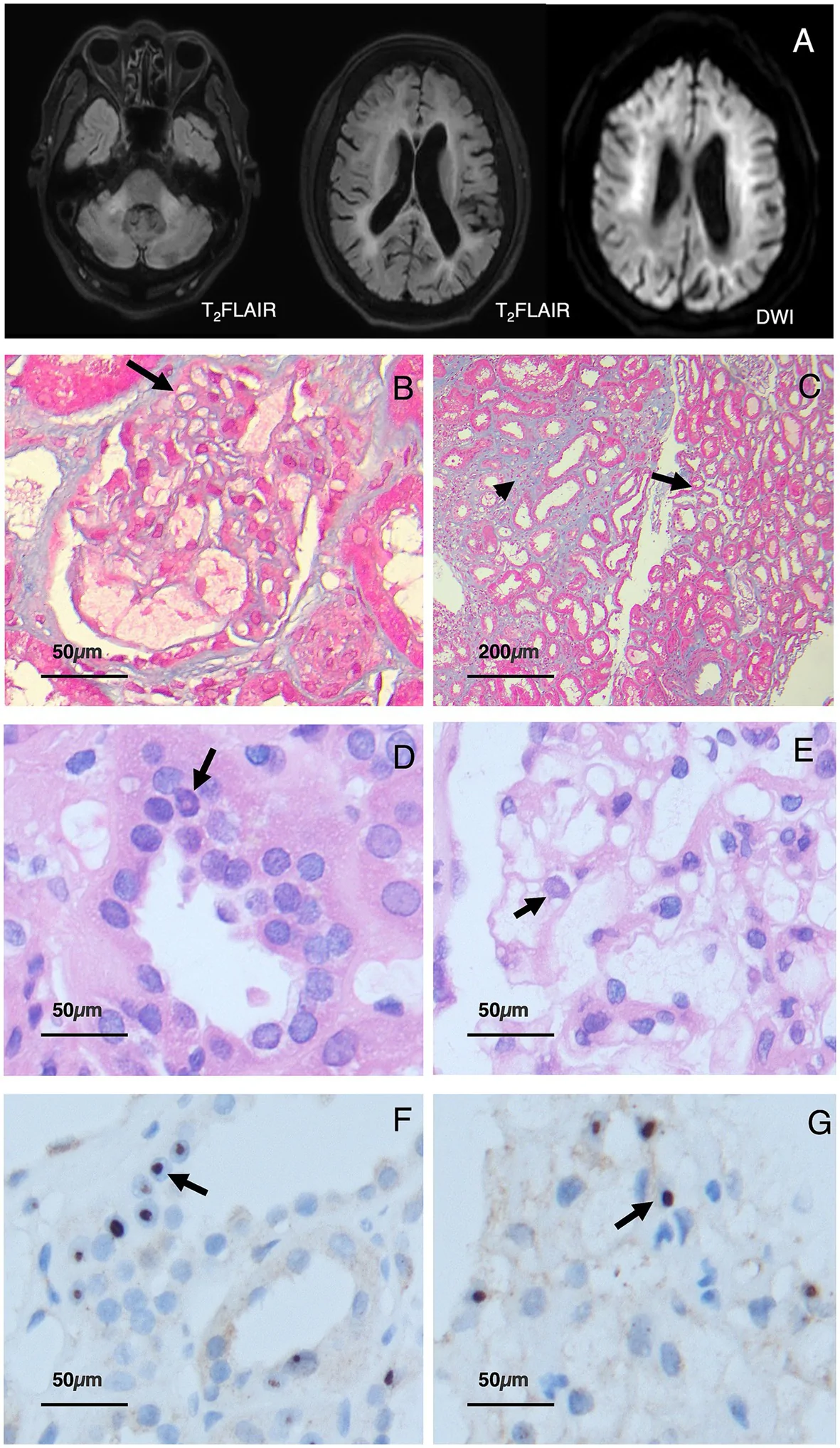
Neuroimaging and renal histopathological findings for Patient 1. **(A)** Brain MRI showed symmetrical pontine and bilateral periventricular white matter lesions on T2-FLAIR, high-intensity signals at the corticomedullary junction on DWI, and cerebral atrophy. **(B)** Renal biopsy (Masson staining, 400×) showed mild glomerular basement membrane thickening (long arrow). **(C)** Renal biopsy (Masson staining, 100×) showed focal tubular atrophy (long arrow) and interstitial fibrosis (arrowhead). **(D,E)** High-magnification views (H&E staining, 400×) revealed eosinophilic hyaline intranuclear inclusions (long arrows) in tubular cells **(D)** and glomerular cells **(E)**. **(F,G)** P62 immunohistochemical staining (400×) confirmed intranuclear inclusions (long arrows) in tubular cells **(F)** and glomerular cells **(G)**.

**Figure 7:**
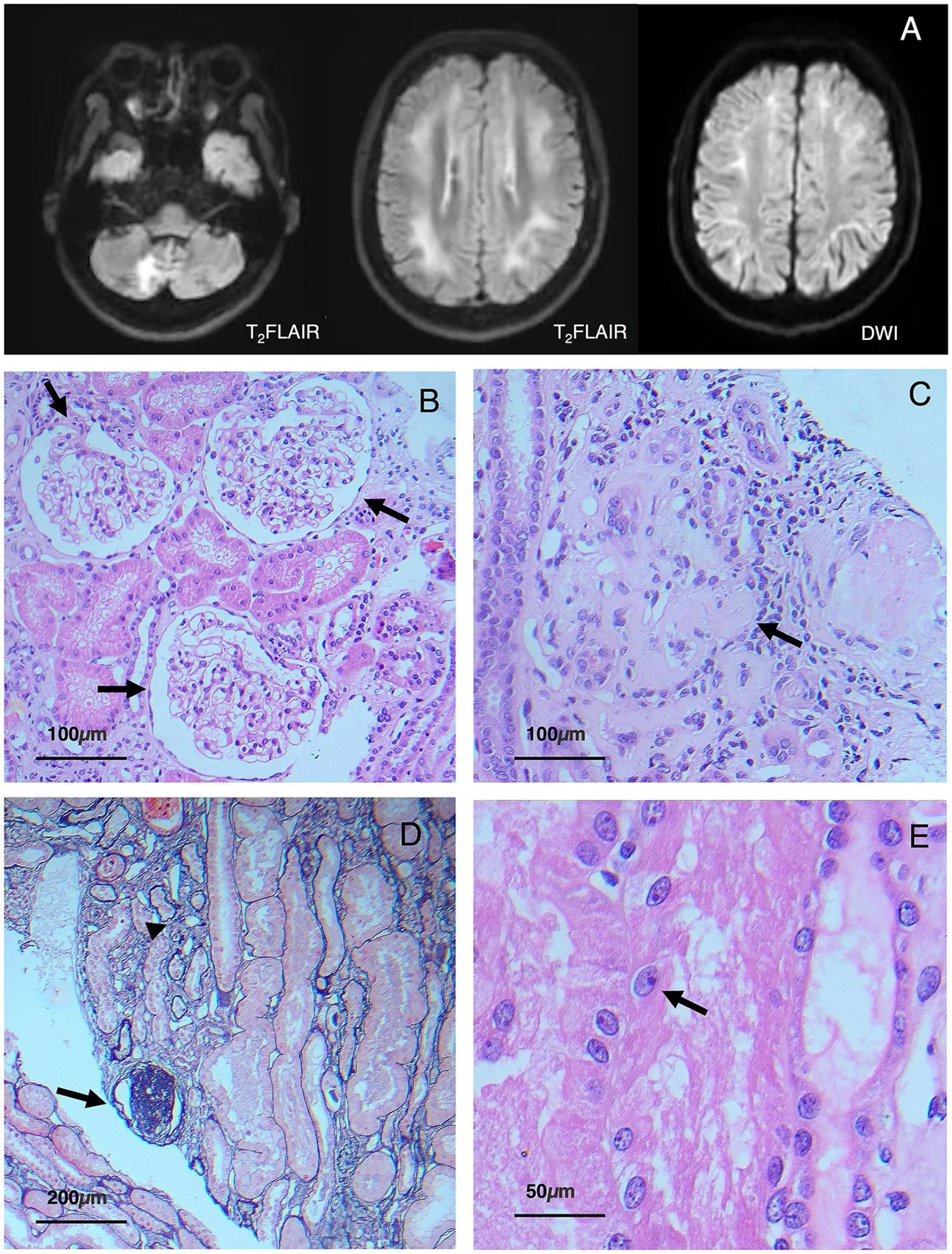
Neuroimaging and renal histopathological findings for Patient 2 **(A)** Brain MRI revealed cerebellar and centrum semiovale white matter lesions on T2-FLAIR and corticomedullary junction hyperintensities on DWI. **(B)** Renal biopsy (H&E staining, 200×) showed three relatively normal glomeruli (long arrows). **(C)** A sclerotic glomerulus (long arrow) was observed (H&E staining, 200×). **(D)** A sclerotic glomerulus (long arrow) and adjacent tubular atrophy (arrowhead) were shown with PAS/PASM and Masson’s trichrome staining (100×). **(E)** High-magnification view (H&E staining, 400×) showed an eosinophilic hyaline intranuclear inclusion (long arrow) in a renal tubular cell.

Following genetic confirmation of NIID (*NOTCH2NLC* GGC repeat expansions of 139 and 121 copies), retrospective analysis of the archival renal biopsies revealed eosinophilic hyaline intranuclear inclusions in tubular cells in both cases and in glomerular cells in one case (Patient 1), which were confirmed by p62 immunostaining (Figures 6, 7). At the time of neurological presentation, renal function was preserved, but significant albuminuria was detected in the first patient (EPI-eGFR of 103.06 mL/min/1.73 m^2^; urinary microalbumin concentration of 1853 mg/L), while the second patient developed mild renal dysfunction (EPI-eGFR of 77.79 mL/min/1.73 m^2^) with persistent proteinuria (urinary microalbumin concentration of 1,347 mg/L).

## Discussion

NIID is a systemic disease, and our study shows that the prevalence of kidney involvement is much higher than previously recognized. In 96 genetically confirmed patients, 20.8% had an eGFR < 90 mL/min/1.73 m^2^. Among those with normal eGFR, 63.6% had abnormal urinary biomarkers. Overall, approximately 75% of patients showed evidence of kidney involvement—substantially higher than reported in previous study (8). This difference may be attributable to our inclusion of five urinary early renal injury markers, which identified a subset of patients with subclinical renal dysfunction who would otherwise have been missed. Nonetheless, as a single-center, cross-sectional study conducted at a national tertiary referral center for neurological diseases, our cohort is likely enriched for patients with more advanced, symptomatic, or multisystem disease. This referral-based ascertainment tends to inflate prevalence estimates, and the direction of the resulting bias is upward. Accordingly, the overall renal-involvement estimate of approximately 75% would perhaps be best viewed as an upper-bound estimate of detectable renal involvement in a hospital-based NIID population, and the true prevalence in community or non-specialized settings is likely lower. The severity of renal involvement in our cohort was heterogeneous. Most patients had subclinical early injury manifesting only as abnormal urinary markers. Among those with reduced eGFR, most were classified as stage G2 (mild decline), although a small proportion progressed to stages G4–G5. In contrast, the previous cohort study by Ji et al. did not report CKD staging (8). These findings underscore that NIID is far from a pure neurological disorder. It has prominent renal involvement, and subclinical renal changes are common.

Urinary multi-parameter biomarkers are sensitive tools for screening early renal dysfunction. Urinary microalbumin, transferrin, immunoglobulin G, and α1-microglobulin showed stepwise significant increases across the three groups whereas eGFR and serum creatinine did not differ between the normal and early injury groups. This indicates that urinary markers can detect early kidney injury even when eGFR remains normal. However, the urinary markers are sensitive indicators of glomerular and tubular injury but are not specific to NIID. They reflect general renal injury rather than NIID-specific pathology. Hypertension, diabetes, and aging are established contributors to renal impairment and may act as independent confounders. To address this concern, we performed a sensitivity analysis restricted to patients without these comorbidities. Notably, the proportion of patients with abnormal urinary biomarkers remained at or above 64% across all comorbidity-free subgroups, and was numerically highest (76.19%) in patients free of both diabetes and hypertension. If hypertension or diabetes were the primary drivers of the urinary abnormalities, we would expect removal of these comorbidities to substantially reduce the abnormal rate and the observed pattern does not support this expectation. These findings suggest that the high frequency of subclinical urinary abnormalities in NIID patients is observed regardless of the presence of diabetes or hypertension. Nevertheless, residual confounding cannot be entirely excluded given the modest sample size and the potential contribution of other age-related factors. The five biomarkers reflect injury at different sites of the nephron by molecular weight. Microalbumin (~66 kDa) and transferrin (~77 kDa) indicate early glomerular injury, immunoglobulin G (~150 kDa) indicates more advanced, non-selective glomerular injury, and α1-microglobulin (~26 kDa) indicates proximal tubular dysfunction (12). α2-macroglobulin (~720 kDa) crosses the glomerular barrier only with the most severe glomerular damage and otherwise reflects a post-renal source (13). The concurrent elevation of both glomerular and tubular markers in our cohort suggests that renal involvement in NIID is a mixed glomerular and tubular process. Among the five markers, urinary microalbumin exhibited the highest discriminative ability in our ROC analysis. This pattern would support its use as a practical first-line screening marker. Urinary α1-microglobulin also performed well and may serve as a complementary marker for detecting proximal tubular injury, which is not captured by microalbumin alone. However, these results should be interpreted with caution. Because early renal involvement was itself defined by the presence of urinary abnormalities, the ROC analyses describe the relative sensitivity of each marker rather than their independent diagnostic accuracy. No combined model was constructed to avoid circularity. In addition, the sample size was modest, which precluded a stable multivariable model. The proposed cutoffs were derived from a single, modest-sized cohort and require external validation. To our knowledge, no previous studies have paid sufficient attention to early urinary kidney injury markers in NIID.

Hypertension was an independent risk factor for declining kidney function and increased the risk of eGFR < 90 mL/min/1.73 m^2^ nearly fourfold (OR = 3.956). This finding identifies hypertension as a potentially modifiable factor worth monitoring. However, the mechanistic link between hypertension and renal injury in NIID warrants further consideration. Autonomic dysfunction is a known feature of NIID (10) and may contribute to both hypertension and renal impairment. Sympathetic overactivity can lead to labile hypertension (14). It can also alter renal hemodynamics, increase renovascular resistance, and promote sodium retention (15). Each of these effects can promote kidney injury. Therefore, autonomic dysfunction could be a common upstream mechanism that contributes to both hypertension and kidney injury. In our cohort, the autonomic dysfunction-predominant subtype was numerically more frequent in the renal dysfunction groups, although the difference was not statistically significant (20% in the renal dysfunction group vs. 17.86% in the early renal dysfunction group and 6.25% in the normal renal function group) (*p* = 0.710). Although the trend is consistent with the hypothesis, the difference was not statistically significant, and this mechanistic model should be considered speculative pending further studies. Sustained hypertension may further compromise renal microvascular autoregulation, thereby aggravating glomerular injury (16, 17). Conversely, progressive renal dysfunction may further elevate blood pressure through volume overload and activation of the renin–angiotensin–aldosterone system (18), suggesting a bidirectional pathogenic cycle. A similar mechanistic link has been described in other neurodegenerative disorders with prominent autonomic involvement. In patients with synucleinopathies (including multiple system atrophy, Parkinson’s disease, and pure autonomic failure) and neurogenic orthostatic hypotension, supine hypertension arising from autonomic failure is associated with lower eGFR and other markers of target organ damage (19). This parallel supports the plausibility of an autonomic mechanism for renal involvement in NIID. A perfusion-related mechanism may also contribute. Prior studies have reported abnormal cerebral perfusion in NIID (20, 21), raising the possibility that comparable perfusion abnormalities occur in the kidney. Renal hypoperfusion could act synergistically with hypertension to accelerate kidney injury. Together, these considerations support an autonomic and vascular mechanism for renal involvement in NIID. However, because of the cross-sectional design, we cannot determine the temporal sequence between hypertension and renal decline. Longitudinal studies incorporating autonomic function testing will be needed to disentangle these pathways. Furthermore, direct evidence from renal perfusion studies is needed to confirm this hypothesis. In contrast, no significant differences were observed in diabetes or glycated hemoglobin levels across renal function categories.

The apparently protective effect of a positive family history (adjusted OR 0.279, 95% CI 0.087–0.896) is counterintuitive and should be interpreted cautiously. We initially attributed it to earlier and more frequent medical surveillance among individuals with affected relatives. This hypothesis received only partial support. Family history-positive patients had significantly more outpatient visits before admission (median 3 vs. 1, *p* = 0.008), but they did not undergo more renal function testing, and the two groups did not differ in age at onset, age at diagnosis, disease duration, or onset-to-diagnosis interval. This indicates that more frequent medical contact did not translate into earlier diagnosis or more intensive renal monitoring, so increased surveillance alone is unlikely to fully explain the association. Another possibility is survivorship bias. Family history-positive patients are often identified through family screening after an affected relative is diagnosed. When the most severely affected member of a pedigree has died or become too ill, the relatives who are enrolled tend to have milder disease and better renal function. By contrast, family history-negative patients are identified by their own symptoms and lack this pedigree-level filtering. This asymmetry could produce an apparent protective association between family history and renal function. A third, albeit related, consideration is that shared genetic and epigenetic backgrounds within families may influence renal susceptibility. GGC repeat number did not differ between family history-positive and -negative patients (114.10 vs. 114.57, *p* = 0.907), suggesting that the protective association is unlikely to be explained by a difference in repeat length between the two groups. However, factors beyond repeat length are known to shape the NIID phenotype, including GGA interruptions within the GGC expansion (22), the repeat-associated translation products polyglycine, polyalanine, and polyarginine (23), and aberrant DNA methylation of the *NOTCH2NLC* promoter, the level of which has been inversely correlated with the extent of multi-systemic involvement (24). Because such genetic and epigenetic modifiers, as well as shared environmental exposures, tend to be transmitted or shared within pedigrees, members of a given family may exhibit a similar pattern and severity of renal involvement. Consistent with this, the three patients from a single family in our cohort were all classified into the same renal function stratum, and a previously reported family showed concordant progression to renal failure with focal segmental glomerulosclerosis on biopsy (25). This familial concordance suggests that family-specific genetic or epigenetic backgrounds may determine the severity of renal involvement. The apparent protective effect of family history may therefore reflect, in part, the enrollment of families that happen to carry comparatively favorable modifying backgrounds, rather than a true protective effect of family history itself. Given the wide confidence interval, the small number of events, and the retrospective design, this protective association should be regarded as hypothesis-generating. Family-based genetic and epigenetic studies with long-term longitudinal follow-up are warranted to elucidate the relationship between family history and renal outcomes in NIID.

Expanded GGC repeats are transcribed into guanine-rich RNA species that accumulate in the nucleus. These aberrant RNAs interact with various RNA-binding proteins, leading to intranuclear inclusion formation and disrupting essential nuclear functions such as transcription and RNA splicing, ultimately contributing to cellular dysfunction and death (2, 26). However, our study found no overall correlation between genetic burden and kidney function. Ji et al. also reported no direct correlation between repeat expansion size and kidney involvement (8). Their study additionally suggested that acquired inflammatory mechanisms critically determine renal phenotype. Our study further confirms that skin inclusion burden, a more intuitive measure of genetic burden, is also not significantly correlated with renal involvement. Nevertheless, it is worth noting that our subgroup analysis found an inverse correlation between eGFR and GGC repeat number in patients with preserved renal function. This suggests that studying the relationship between genetic burden and kidney function may require focusing on patients with completely normal kidney function before the early injury stage appears. At this stage, a direct toxic effect of the genetic burden may exist, potentially through disruption of normal cellular function (1, 27). The loss of this correlation in patients with established renal impairment suggests that secondary mechanisms may become the main drivers of disease progression once a critical injury threshold has been crossed. These secondary mechanisms may include inflammatory responses, fibrosis, and vascular changes (28–30). The lack of a consistent association between genetic or inclusion burden and renal impairment implies that treatments targeting modifiable non-genetic factors, such as immune or vascular pathways, could help reduce kidney injury in NIID patients.

Kidney pathology can predate neurological symptoms by years and may be misdiagnosed as other glomerular diseases. Consistent with prior case reports (6, 7, 25, 31), our study confirmed the presence of intranuclear inclusions in kidney-specific cells. While most earlier studies documented inclusions predominantly in tubular epithelial cells (6, 7, 31), our findings align more closely with those of Watanabe et al. (25), as we also detected inclusions in both tubular and glomerular compartments. In contrast, Morita et al. reported inclusions exclusively in interstitial fibroblasts (32). Collectively, these observations indicate broad cellular vulnerability to intranuclear inclusion formation within the kidney. Furthermore, renal tubules are likely the best place to look for intranuclear inclusions on kidney biopsy. However, urinary markers of glomerular dysfunction are more sensitive for detecting early kidney injury in NIID. This suggests that intranuclear inclusion burden on kidney pathology may not directly correlate with kidney function decline. This possibility requires further study.

Our findings have immediate clinical implications. Renal function assessment, including urinary biomarkers, should be incorporated into the standard management of NIID patients. Patients with hypertension had more severe kidney function impairment, highlighting the importance of blood pressure control in NIID patients. Furthermore, in cases of unexplained proteinuria or glomerulopathies, particularly those accompanied by subtle neurological manifestations or a family history of neurological disorder, pathologists are encouraged to include NIID in the differential diagnosis and actively search for intranuclear inclusions within renal biopsy specimens. This evaluation can be enhanced by p62 immunohistochemical staining, as renal tubules represent the most frequent and accessible site for detecting inclusions.

Several limitations should be acknowledged. The single-center, tertiary referral design introduces a selection bias that may overestimate the prevalence of renal involvement. Multicenter, population-based studies with standardized urinary testing are needed to provide generalizable prevalence estimates. The sample size, although substantial for a rare disease, remains relatively small. The cross-sectional design precludes assessment of causality or disease progression over time. The absence of a disease-control group is a genuine limitation and that common comorbidities including hypertension and diabetes are potential confounders. Although our sensitivity analysis partially mitigates this concern, it does not completely eliminate the possibility of residual confounding from these or other age-related factors. Future studies incorporating age- and comorbidity-matched healthy controls or patients with other neurodegenerative diseases are needed to establish the NIID-specificity of these urinary markers. Renal biopsy specimens were available for only two illustrative cases rather than the entire cohort, limiting the generalizability of the pathological findings. Important questions remain unanswered. Longitudinal studies are needed to determine the natural history and progression rate of NIID-associated nephropathy. The precise molecular mechanisms through which *NOTCH2NLC* GGC repeat expansions lead to renal cell dysfunction and inclusion formation require investigation using cellular and animal models. Future studies with larger sample sizes and serial renal biopsies could help clarify the evolution of pathological changes and their correlation with clinical progression.

## Conclusion

In summary, renal involvement is common in NIID, with detectable abnormalities in approximately 75% of patients in our hospital-based cohort, though most cases are subclinical. Sensitive urinary biomarkers may aid early detection of renal involvement before eGFR declines. In our analysis, microalbumin showed the highest discriminative ability for glomerular injury, complemented by α1-microglobulin for tubular injury, although the proposed cutoffs require external validation before clinical use. Hypertension was an independent, potentially modifiable risk factor for reduced eGFR, underscoring the importance of blood pressure management in NIID patients. GGC repeat number and skin inclusion burden did not correlate with renal function overall, suggesting that kidney injury in NIID may be driven by mechanisms independent of simple genetic or pathological burden measures. For patients with unexplained renal impairment, renal biopsy examination for intranuclear inclusions may provide diagnostic clues for underlying NIID. This study supports early detection, risk factor management, and multidisciplinary care in NIID. Future research should focus on tissue-specific mechanisms of renal injury.

## Data Availability

The raw data supporting the conclusions of this article will be made available by the authors, without undue reservation.
