# Supplementary material for: High burden of kidney involvement in neuronal intranuclear inclusion disease
Source: Front Neurol. 2026 Aug 11;17:1905689. doi: 10.3389/fneur.2026.1905689 (PMC13503263; doi:10.3389/fneur.2026.1905689)
Supplement: Supplementary file 1 [file Data_Sheet_1.PDF]

Supplemental table 1. Multiple linear regression analysis of factors associated with EPI- eGFR

Variable	B (95% CI)	*P*
Age at admission, years	-0.726 (-1.534 to 0.083)	0.077
Age at onset, years	-9.868 (-20.354 to 0.618)	0.065
Hypertension (yes vs no)	5.249 (-5.131 to 15.630)	0.315
Family history (yes vs no)	0.238 (-0.404 to 0.880)	0.460
GGC repeat number	-0.008 (-0.278 to 0.263)	0.956
Intranuclear inclusion count	-130.837 (-384.517 to 122.842)	0.305

Model: $R^2 = 0.175$, adjusted $R^2 = 0.074$, $P = 0.134$. GGC repeat number: number of *NOTCH2NLC* GGC repeat expansion

Supplemental table 2. Tertile-based analysis of EPI-eGFR by GGC repeat number and intranuclear inclusion count

Variable	Tertile group	N	eGFR (mL/min/1.73m²), mean (SD)	ANOVA *P*	Linear trend *P*	Kruskal-Wallis *P*
GGC repeat number	Low (≤P33)	32	94.90 (21.69)			
	Middle (P33–P67)	33	96.53 (15.31)	0.870	0.851	0.955
	High (≥P67)	31	93.93 (22.33)			
Intranuclear inclusion count	Low (≤P33)	19	94.59 (22.72)			
	Middle (P33–P67)	18	99.51 (8.15)	0.654	0.923	0.943
	High (≥P67)	19	93.97 (24.12)			

Tertiles were defined based on the 33rd and 67th percentiles of each variable. Linear trend test was performed using polynomial contrast. Kruskal-Wallis test was used as a nonparametric alternative. GGC repeat number: number of *NOTCH2NLC* GGC repeat expansion

Supplemental Table 3. Clinical characteristics and healthcare monitoring indicators stratified by family history

	**Family history-positive (n=49)**	**Family history-negative (n=47)**	**Z/χ²/t**	***P***
Age at onset, years, M (Q1, Q3)	57.00 (48.00, 61.00)	58.00 (50.00, 62.50)	Z=-0.870	0.385
Age at diagnosis, years, M (Q1, Q3)	63.00 (58.00, 66.00)	64.00 (59.00, 66.50)	Z=-0.694	0.488
Disease duration, years, M (Q1, Q3)	5.50 (3.00, 10.00)	5.00 (2.00, 8.50)	Z=-1.525	0.127
Onset-to-diagnosis interval, years, M (Q1, Q3)	5.00 (2.00, 12.00)	5.00 (1.75, 7.50)	Z=-1.150	0.250
Outpatient visits before admission, n, M (Q1, Q3)	3.00 (1.00, 3.50)	1.00 (1.00, 2.00)	Z=-2.658	**0.008**
Renal function tests before admission, n, M (Q1, Q3)	1.00 (1.00, 2.00)	1.00 (1.00, 2.00)	Z=-0.880	0.379
Hypertension, n (%)	22 (44.90%)	26 (55.32%)	χ^2=1.042	0.307
ARB/ACEI use (among hypertensive patients), n (%)	9 (40.91%)	10 (38.46%)	χ^2=0.030	0.863
Regular antihypertensive use (among hypertensive patients), n (%)	14 (63.64%)	17 (65.38%)	χ^2=0.016	0.900
GGC repeat number, mean (SD)	114.10 (19.31)	114.57 (20.41)	t=−0.117	0.907

M (Q1, Q3): median (interquartile range); SD: standard deviation; ARB: angiotensin receptor blocker; ACEI: angiotensin-converting enzyme inhibitor; GGC repeat number: number of *NOTCH2NLC* GGC repeat expansion
